# Novel KMT2B gene mutation in MUC4 positive low-grade fibromyxoid sarcoma

**DOI:** 10.1186/s13000-024-01458-5

**Published:** 2024-02-12

**Authors:** Liying Zhang, Luqiao Luo, Chao Liu, Zhi Li

**Affiliations:** 1 Department of Pathology, Cancer Hospital of Shantou University Medical College, Shantou, China; 2 Department of Pathology, Guangdong Provincial People’s Hospital, Guangdong Academy of Medical Sciences, Guangzhou, China

**Keywords:** Low-grade Fibromyxoid Sarcoma, KMT2B, MUC4, FUS

## Abstract

**Background:**

Low-grade Fibromyxoid Sarcoma(LGFM)is a rare fibrosarcoma, which mainly occurs in young people and is mostly seen in the trunk and limbs. The tumor is usually FUS-CREB3L2 fusion caused by t(7;16)(q32-34;p11)chromosome translocation, and rarely FUS-CREB3L1 and EWSR1-CREB3L1 fusion. MUC4 diffuse strong positive can be used as a specific index of LGFM. LGFM is similar to Sclerosing Epithelioid Fibrosarcoma(SEF) and may have the same origin.

**Case presentation:**

We report a case of LGFM in the chest wall. A female who is 59 years old. In 2016, CT showed dense nodule shadow and focal thickening of the left pleura, the patient underwent surgery, Pathological report that low to moderate malignant fibrosarcoma(fibromyxoid type). The CT re-examination in 2021 showed that the tumors on the left chest wall were significantly larger than before. Pathological examination showed the disease is composed of alternating collagen like and mucinous areas. Under high-power microscope, the tumor cells are consistent in shape, spindle or short spindle, and the tumor cells are arranged in bundles. In local areas, the density of tumor cells is significantly increased, mixed with collagen fibers, and small focal SEF appear. The result of immunohistochemistry showed that SMA, Desmin, CD34, STAT6, S100, SOX10, HMB45 and Melan A were negative, EMA was weakly positive, MUC4 was diffuse and strongly positive, and Ki67 index was low (3%).

**Conclusion:**

Sequencing results showed that MET, EGFR, KMT2B and RET gene were mutated in LGFM, and KMT2B gene had cancer promoting effect, but there was no literature report in LGFM, which may be of certain significance for the diagnosis and treatment of LGFM.

**Supplementary Information:**

The online version contains supplementary material available at 10.1186/s13000-024-01458-5.

## Introduction

LGFM is a rare subtype of fibrosarcoma. It mainly occurs in young people, and the proportion of men and women is not different [1, 2]. The tumor was first proposed by Evans in 1987 [3]. Sarcomas account for 1% of cancers, while LGFM is estimated to account for less than 5% of soft tissue sarcomas [1]. The tumor mainly appears in the trunk and limbs [4]. However, the tumor can be seen in the head and neck, mediastinum, small intestine and scrotum [5–8].

LGFM is composed of alternately distributed collagen like and mucinous areas. The cells are mainly spindle cells, with round or oval nuclei, light eosinophilic cytoplasm and unclear boundary. They frequently show mild nuclear atypia and mitotic activity. They are often arranged in a spiral, linear or disorderly distribution. Although the tumor looks mild in morphology, it can have local recurrence and distant metastasis [1, 9–13].

Chromosome translocation is found in about 20–25% of sarcomas [14], but for LGFM patients, chromosome translocation basically occurs. The most common is t(7; 1)(q32-34; p11) chromosome translocation, resulting in FUS-CREB3L2 fusion [15–17]. FUS-CREB3L2 fusion was first found in LGFM by Storlazzi, et al [18]. FUS-CREB3L2 fusion accounted for about 90% of all patients. Other rare fusions are FUS-CREB3L1 and EWSR1-CREB3L1 [4, 15, 19].

Fused in Sarcoma(FUS) is a widely expressed protein, RNA-DNA binding protein, mainly expressed in the nucleus of cells [20].It consists of N-terminal and C-terminal. The N-terminal has a transcriptional activation domain and the C-terminal contains RNA recognition motif(RRM) [21].The FUS regulates DNA repair transcription in the nucleus, RNA splicing, and its export to the cytoplasm [20]. The FUS has several phosphorylation sites, and interestingly, the FUS contains two EGFR-targeted phosphorylation sites [22].

It has been reported that MUC4 is a specific and sensitive indicator of LGFM [23]. It is worth noting that MUC4 expression is also found in SEF, ossifying fibromyxoid tumor, synovial sarcoma, myoepithelial carcinoma and epithelioid gastrointestinal stromal tumor [23, 24]. However, MUC4 negative LGFM with FUS-CREB3L2 fusion has been reported [25].

Here, we report a case of LGFM appearing in the chest wall, which has diffused strong MUC4 positive in immunohistochemistry, FISH analysis show that the FUS gene is abnormal. We sequenced and found that the tumor was mutated in four genes: MET, EGFR, KMT2B and RET.

## Case report

### Patient information and medical history

A female who is 59 years old. In 2016, CT showed dense nodule shadow, high-density lock strip shadow and fuzzy patch shadow in the left lower lung, and focal thickening of the left pleura. Therefore, “left chest wall tumor resection + left lower lung tumor resection” was performed. Pathological report showed that low to moderate malignant fibrosarcoma(fibromyxoid type). In 2018, a peanut sized tumor was found on the left chest wall, which was hard without tenderness and untreated. CT Reexamination in 2020 showed multiple solid nodules in both lungs with clear boundary; The left chest wall is a kind of round mass shadow, which seems to have a pedicle connected with the rear muscle and is unevenly strengthened. It increased like an egg within one year. The CT re-examination in 2021 showed that there were multiple solid nodules in both lungs and tumors on the left chest wall, and the tumors on the left chest wall were significantly larger than before. No abnormality was found in other examinations.

### Gross morphology and pathological diagnosis

**Fig. 1:**
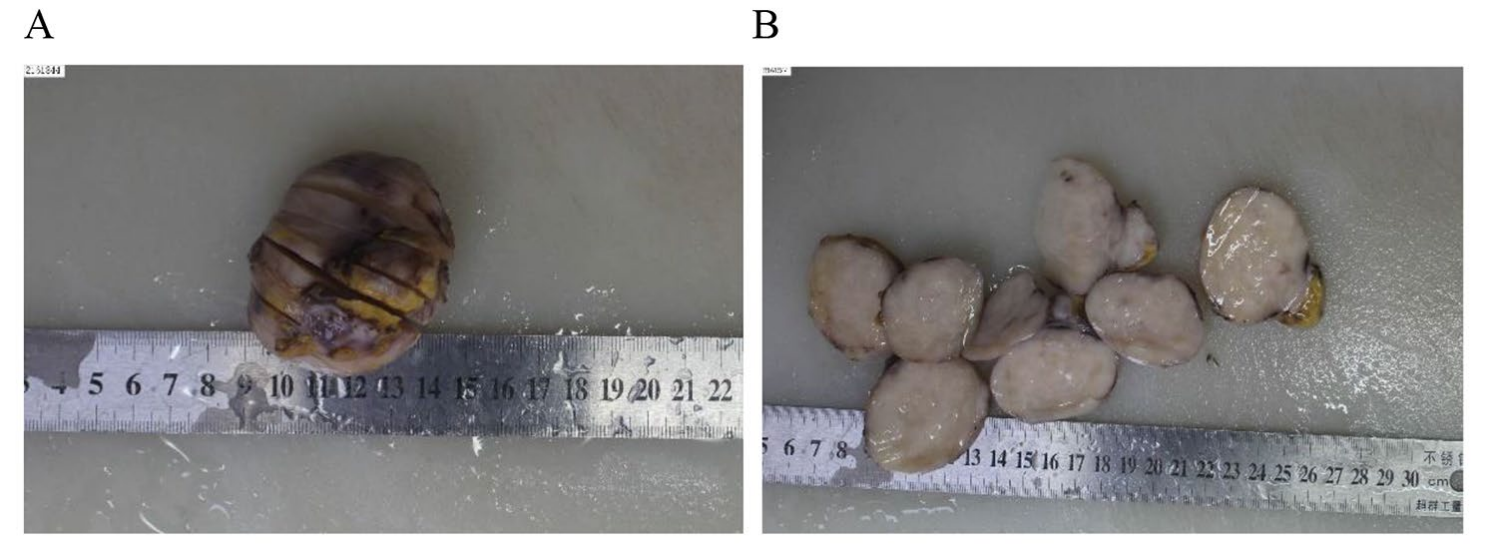
The boundary of chest wall tumor is clear **(A)** and the section is grayish white and locally grayish yellow **(B)**

**Fig. 2:**
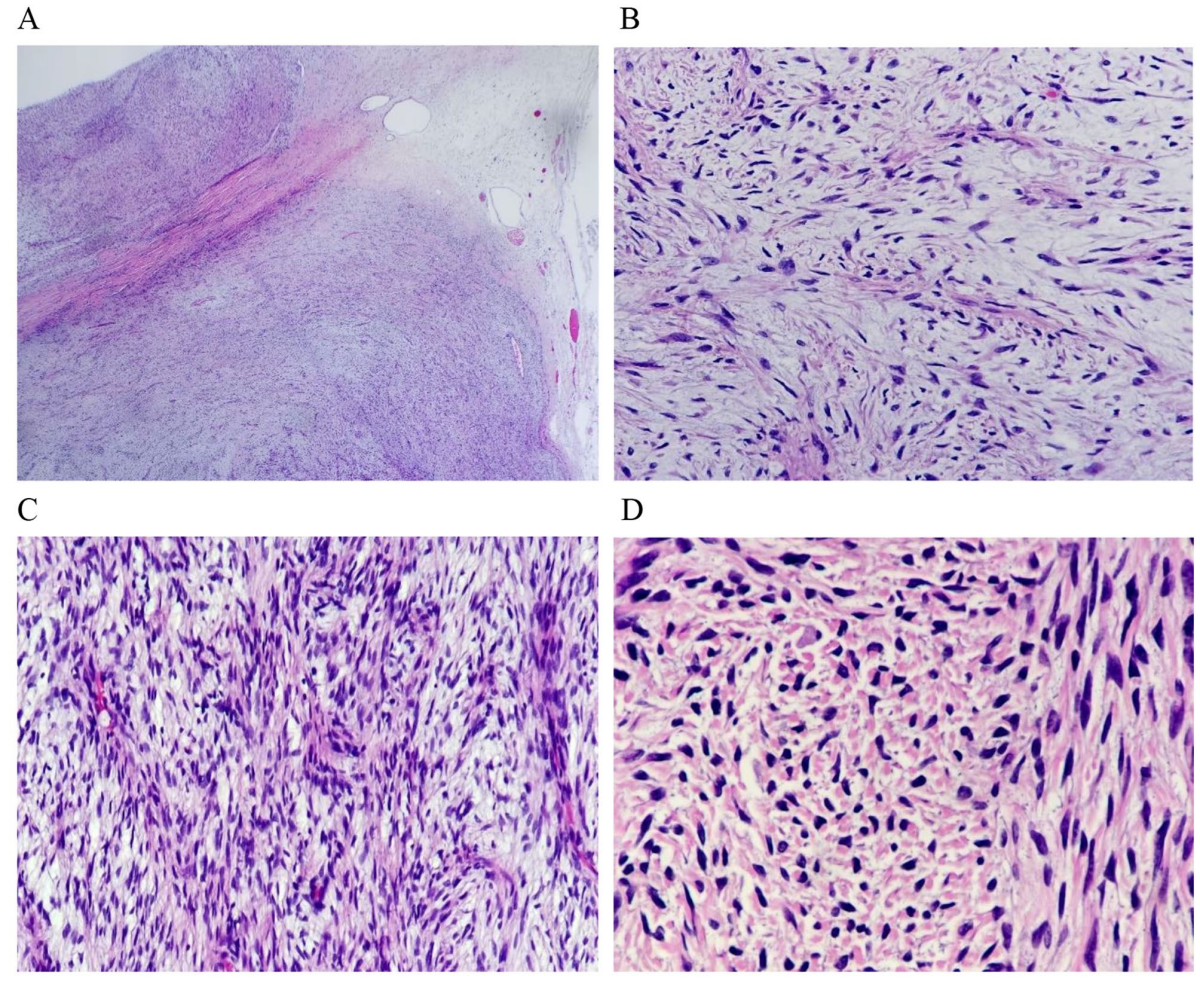
LGFM appears to be encapsulated and slightly leafy at low magnification **(A)**. At high magnification, mixed migration of collagen like area and mucoid area **(B)**, tumor cells are consistent in morphology, showing spindle or short spindle shape **(C)**. SEF appeared in small foci **(D)**

General observation, the tumor is intact and well-defined, with a size of 6 × 5 × 4 cm, its texture is grayish white, with local areas showing alternating yellow and white(Fig. 1). Although the tumor generally seems to have a clear boundary, it infiltrates into adjacent tissues under microscope. At low magnification, we can see that the tumor is composed of alternating collagen like and mucinous areas. There is migration or transition between the two areas, and we can also see a relatively clear boundary. At high magnification, the tumor cells are consistent in morphology, spindle or short spindle, and star shaped in the myxoid area, similar to fibroblasts. The nucleus is round or oval, deeply stained, and the chromatin is evenly distributed. The mitotic nucleus is not obvious; The cytoplasm was lightly stained and the cell boundary was unclear. The tumor cells are arranged in bundles, linear arrangement or disorderly distribution. The blood vessels in tumor cells are relatively rare, mostly arched, curved or arc-shaped. In myxoid areas, sometimes branching capillary networks similar to myxoid liposarcoma can be seen. In the local area, the density of tumor cells increased significantly, mixed with collagen fibers, and small focal sclerosing epithelioid fibrosarcoma appeared(Fig. 2).

**Fig. 3:**
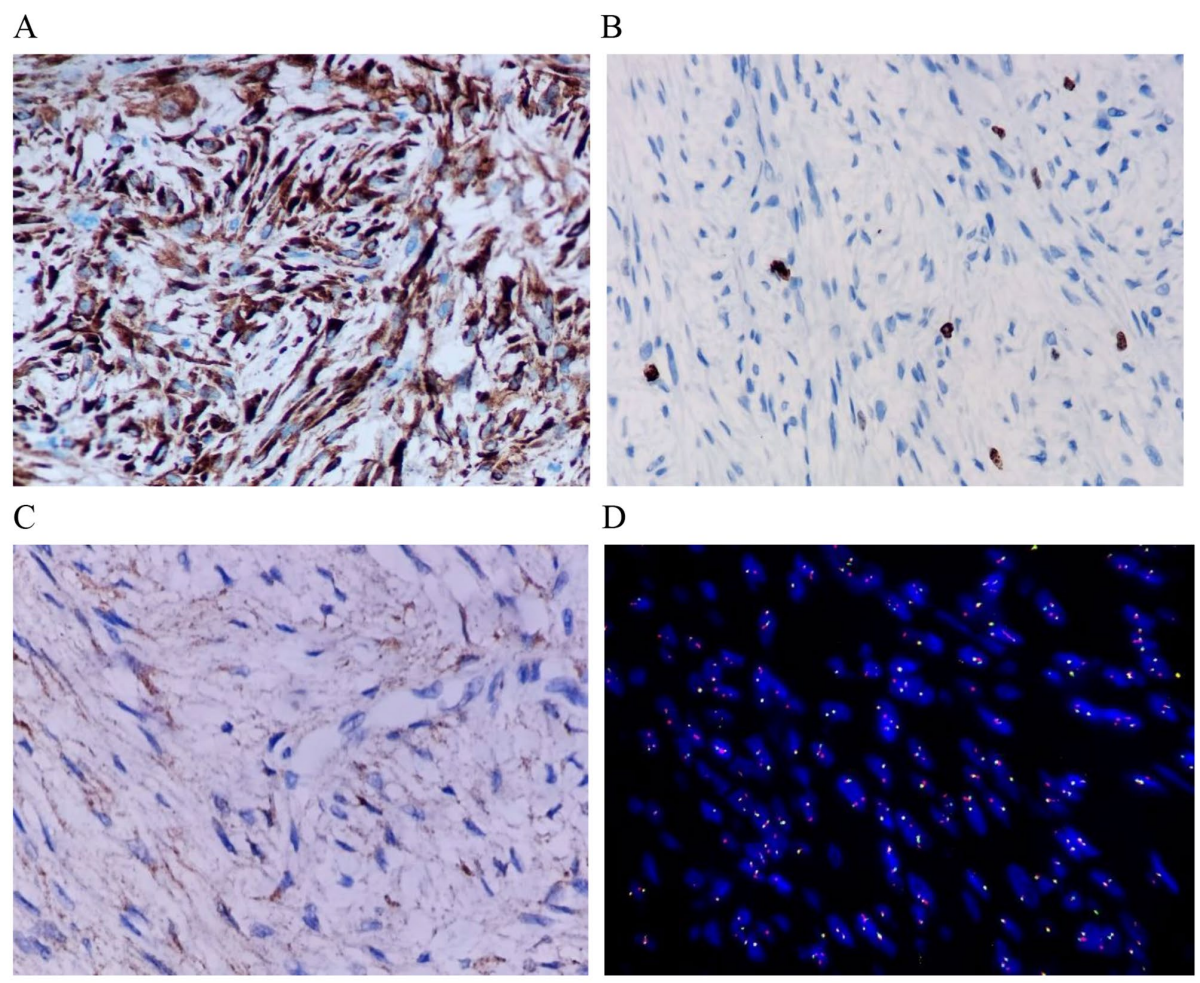
Immunohistochemistry show MUC4 diffuse strong positive **(A)**, Ki67 index low **(B)** and EMA Focal positive **(C)**. FISH results FUS gene break mutation **(D)**

Immunohistochemical results showed that SMA, Desmin, CD34, STAT6, S100, SOX10, HMB45 and Melan A were negative, EMA was weakly positive, MUC4 was diffuse and strongly positive, and Ki-67 index was low(3%). FISH results showed that DDIT3 gene mutation was not found, but FUS gene was break mutation(Fig. 3).

### Next-generation sequencing technology

In order to find out whether there are mutations in other genes in low-grade fibromyxoid sarcoma except FUS-CREB3L2 fusion, FUS-CREB3L1 and EWSR1- CREB3L1 fusion [4, 15, 18, 19]. Subsequently, we conducted next-generation sequencing experiment, which showed that there were mutation in four gene MET, EGFR, KMT2B and RET(Table 1). It is noteworthy that KMT2B mutation in Low-grade fibromyxoid sarcoma has not been reported.

KMT2B belongs to a member of the histone lysine N-methyltransferase 2 family [26]. KMT2 is divided into KMT2A, KMT2B, KMT2C, KMT2D, etc. of which the two have high homology (KMT2A and KMT2B, KMT2C and KMT2D). The first four are the most common gene changes in tumor types, among which the mutation rate is higher in melanoma, Endometrial carcinoma and lung cancer. The mutation frequencies of single genes in KMT2 family are KMT2D(18%), KMT2C(15%), KMT2A(9%)and KMT2B(8%) [27].

KMT2B, also known as mixed lineage leukemia 2(MLL2), OMIM 606,834, is located on chromosome 19q13 12 [28], which has a similar gene structure to MLL1(KMT2A)located on chromosome 11q23 [27]. The structural components of the gene include the catalytically active C-terminal SET domain, a CXXC domain, an AT hook and several plant homeotic domains(PHD)in the N-terminal region [27, 29, 30]. The CXXC recognizes and binds to non-methylated CpG DNA, being critical for the association of MLL2 to chromatin [31],CXXC recognizes CpG islands [32]of most promoters, and PDH is next to zinc finger(ZF)-CXXC, including PDH1-PDH4 [33], PDH possess a Cys4-His-Cys3 motif, mediating binding to methylated histone H3 [34], Although all MLL families contain PDH, PDH3 of MLL2 show different specificity, which mainly binds to H3K4me3 tails [34].The C-terminal SEF domain of KMT2B forms a complex with WRAD, host cell factors 1/2(HCF 1/2)and Menin [30, 35–37], the complex is responsible for the binding of PDH3 and H3K4me3, regulating bivalent developmental genes as well as stem cell and germinal cell Differentiation gene sets [32].In addition, MLL2 plays a key role in embryonic development, the deletion of MLL2 is related to early growth retardation, neural tube defects and apoptosis leading to embryonic death [38–40]. At the same time, MLL2 also has cancer promoting effects, including in colorectal cancer [41], gastric cancer [42], glioblastoma [43], etc.

Epidermal growth factor receptor(EGFR) is a transmembrane glycoprotein. EGFR play key mediators in cell signaling pathways such as proliferation, apoptosis, angiogenesis and metastasis [44]. It has been reported that FUS contains two targeted EGFR phosphorylation sites, mainly Y6 and Y304 in the FUS, suggesting that FUS can be phosphorylated by EGFR, which promoting FUS phosphorylation and inducing nuclear translocation through activated EGFR, so that FUS can mediate collagen production and creates a collagenous background. Therefore, EGFR-mediated FUS phosphorylation regulates FUS nuclear translocation and promotes transcription of fibrotic collagen genes [22].

**Table 1:** The result of Next-generation sequencing

	Tumor specific mutation	
MET	Copy number amplification	-
EGFR	Copy number amplification	-
KMT2B	Missense mutation in exon 28 of p.F2163Y	c.6488T > A (p.F2163Y)
RET	Missense mutation in exon 20 of p.D1113G	c.3338A > G (p.D1113G)

## Conclusion

Our case is the first to report the mutation of KMT2B gene in MUC4 diffusely positive low-grade fibromyxoid sarcoma. The tumor morphology is easy to be confused with low-grade malignant myxofibrosarcoma, invasive fibromatosis and sclerosing epithelioid fibrosarcoma, but the current view is that LGFM and SEF may belong to the same source. At low magnification, the tumor is composed of alternating collagen like and mucinous regions, there is migration or transition between the two regions. At the same time, MUC4 is diffuse and strongly positive. It is reported that most LGFM have FUS-CREB3L2 fusion [15–17]. However, we sequenced the case and found mutations in KMT2B, MET, EGFR and RET gene, while no mutations in KMT2B were reported in LGFM before. Meanwhile, it has been reported that EGFR can promote FUS phosphorylation and nuclear translocation, leading to the production of fibrotic collagen, while under microscope, LGFM shows the distribution of gelatinous and myxoid regions alternately which may be related to the simultaneous mutation of these two genes, it needs to be proved later. Meanwhile, the mutation of KMT2B may be a new mutation point of LGFM, which may provide help for the diagnosis and treatment of LGFM.

## Discussison

LGFM is a rare fibrosarcoma, which is mainly found in the limbs and trunk [4], but it also occurs occasionally in the scrotum, mediastinum, head and neck [5, 6, 8], while we report that it occurs occasionally in the chest wall and lung. The tumor is mainly composed of alternating collagen like and mucinous areas. The cells are spindle shaped, the cell density is not high, and it seems mild. In fact, the recurrence and distant metastasis rate is high. It needs to be distinguished from spindle cell tumors and tumors with fibromyxoid stroma, such as low-grade myxofibrosarcoma, neurofibroma, protuberant cutaneous fibrosarcoma, etc. [1, 45]. This case is easy to be mixed with low-grade myxofibrosarcoma because of their similar morphology. LGFM has basically no expression except MUC4 diffuse strong positive, but low-grade myxofibrosarcoma will have DDIT3 gene breakage, while LGFM has MUC4 strong positive expression and FUS-CREB3L2 fusion.

MUC4 is currently considered to be the most specific and sensitive indicator of LGFM. It is diffusely and strongly positive in LGFM, but it is also reported in the literature that MUC4 of LGFM is negative, FUS-CREB3L2 fusion exists at this time [25]. The tumor is also difficult to distinguish from SEF. They are considered to be homologous [46], and the positive expression rate of MUC4 is very high. However, there are also MUC4 negative cases in SEF. YAP1-KMT2A fusion and KMT2A-VIM fusion exist in SEF with MUC4 negative [47].We mentioned earlier that KMT2A and KMT2B belong to the same family, and they have high homology. However, we found the mutation of KMT2B gene in the sequencing of this case, and there are a few other gene fusion in LGFM, which needs to be further verified.

It has been previously reported that EGFR activation can promote FUS phosphorylation and nuclear translocation, and promote transcription of fibrotic collagen genes [22], and the LGFM showed gelatinous and myxoid regions under microscope, at the same time, our result of sequencing and FISH found mutations in both EGFR and FUS, so we speculated that the collagenous background may be related to mutations in EGFR and FUS. Since FUS-CREB3L2 fusion occurs after FUS fracture [18], it cannot be ruled out that FUS mutant fracture may refuse with KMT2B or EGFR or other genes, and this needs to be further proved.

Our sequencing found that there is a mutation of KMT2B gene in LGFM. It is necessary to find out the fusion gene to provide further diagnosis and treatment for the tumor.

### Electronic supplementary material

Below is the link to the electronic supplementary material.

Supplementary Material 1

Supplementary Material 2

## Data Availability

The dataset supporting the conclusions of this article is included within the article.

## Abbreviations

LGFM: Low-grade Fibromyxoid Sarcoma
SEF: Sclerosing Epithelioid Fibrosarcoma
CT: computed tomography
KMT2B: mixed lineage leukemia
EGFR: Epidermal growth factor receptor
MUC4: Mucin-4
MET: Mesenchymal-epithelial transition factor
RET: rearranged during transfection
FUS: Fused in Sarcoma
EMA: epithelial membrane antigen
WRAD: WDR5, RbBP5, ASH2L, ASH2L
