# Supplementary material for: Novel KMT2B gene mutation in MUC4 positive low-grade fibromyxoid sarcoma
Source: Diagn Pathol. 2024 Feb 12;19:30. doi: 10.1186/s13000-024-01458-5 (PMC10860237; doi:10.1186/s13000-024-01458-5)
Supplement: Supplementary file 2 — Supplementary Material 2 [file 13000_2024_1458_MOESM2_ESM.docx]

Novel KMT2B Gene Mutation in MUC4 Positive Low-grade Fibromyxoid Sarcoma

Liying Zhang^1,2^, Luqiao Luo^1^, Chao Liu^1^, Zhi Li^1*^

1 Department of Pathology, Guangdong Provincial People’s Hospital, Guangdong Academy of Medical Sciences, Guangzhou, China

2 Department of Pathology, Cancer Hospital of Shantou University Medical College, Shantou, China

* Corresponding author

**Abstract**

Background

Low-grade Fibromyxoid Sarcoma (LGFM)is a rare fibrosarcoma, which mainly occurs in young people and is mostly seen in the trunk and limbs. The tumor is usually FUS-CREB3L2 fusion caused by t(7;16)(q32-34;p11)chromosome translocation, and rarely FUS-CREB3L1 and EWSR1-CREB3L1 fusion. MUC4 diffuse strong positive can be used as a specific index of LGFM. LGFM is similar to Sclerosing Epithelioid Fibrosarcoma(SEF)and may have the same origin.

Case presentation

We report a case of LGFM in the chest wall. A female which is 59 years old. In 2016, CT showed dense nodule shadow and focal thickening of the left pleura, the patient underwent surgery, Pathological report that low to moderate malignant fibrosarcoma (fibromyxoid type). The CT re-examination in 2021 showed that the tumors on the left chest wall were significantly larger than before. Pathological examination showed the disease is composed of alternating collagen like and mucinous areas. Under high-power microscope, the tumor cells are consistent in shape, spindle or short spindle, and the tumor cells are arranged in bundles. In local areas, the density of tumor cells is significantly increased, mixed with collagen fibers, and small focal sclerosing epithelioid fibrosarcoma(SEF)appear. The result of immunohistochemistry showed that SMA, Desmin, CD34, STAT6, S100, SOX10, HMB45 and Melan A were negative, EMA was weakly positive, MUC4 was diffuse and strongly positive, and Ki67 index was low (3%).

Conclusion

Sequencing results showed that MET, EGFR, KMT2B and RET gene were mutated in LGFM, and KMT2B gene had cancer promoting effect, but there was no literature report in LGFM, which may be of certain significance for the diagnosis and treatment of LGFM.

**Keywords** Low-grade Fibromyxoid Sarcoma, KMT2B, MUC4, FUS

**Introduction**

LGFM is a rare subtype of fibrosarcoma. It mainly occurs in young people, and the proportion of men and women is not different [1, 2]. The tumor was first proposed by Evans in 1987[3]. Sarcomas account for 1% of cancers, while LGFM is estimated to account for less than 5% of soft tissue sarcomas [1]. The tumor mainly appears in the trunk and limbs[4]. However, the tumor can be seen in the head and neck, mediastinum, small intestine and scrotum[5-8].

LGFM is composed of alternately distributed collagen like and mucinous areas. The cells are mainly spindle cells, with round or oval nuclei, light eosinophilic cytoplasm and unclear boundary. They are often arranged in a spiral, linear or disorderly distribution. The cellular atypia is not obvious, and the mitotic image is not easy to see. Although the tumor looks mild in morphology, it can have local recurrence and distant metastasis[1, 9-13].

Chromosome translocation is found in about 20-25% of sarcomas[14], but for LGFM patients, chromosome translocation basically occurs. The most common is t(7; 1)(q32-34; p11)chromosome translocation, resulting in FUS-CREB3L2 fusion[15-17]. FUS-CREB3L2 fusion was first found in LGFMS by Storlazzi, et al[18]. FUS-CREB3L2 fusion accounted for about 90% of all patients. Other rare fusions are FUS-CREB3L1 and EWSR1-CREB3L1[4, 15, 19].

Fused in Sarcoma (FUS)is a widely expressed protein, RNA-DNA binding protein, mainly expressed in the nucleus of cells [20]. It consists of N-terminal and C-terminal. The N-terminal has a transcriptional activation domain that produces fusion proteins after chromosomal translocation, which can cause sarcoma and leukemia, and the C-terminal contains RNA recognition motif (RRM). Arg-Gly-Gly(RGG)repeats abundantly, and Zinc Finger domains are involved in RNA processing[21]. The FUS regulates DNA repair transcription in the nucleus, RNA splicing, and its export to the cytoplasm [20]. The FUS has several phosphorylation sites, and interestingly, the FUS contains two EGFR-targeted phosphorylation sites[22].

It has been reported that MUC4 is a specific and sensitive indicator of LGFM[23]. It is worth noting that MUC4 expression is also found in SEF, ossifying fibromyxoid tumor, synovial sarcoma, myoepithelial carcinoma and epithelioid gastrointestinal stromal tumor [23, 24]. However, MUC4 negative LGFM with FUS-CREB3L2 fusion has been reported [25].

Here, we report a case of LGFM appearing in the chest wall, which has diffuse strong MUC4 positive in immunohistochemistry, FISH analysis show that the FUS gene is abnormal. We sequenced and found that the tumor was mutated in four genes: MET, EGFR, KMT2B and RET.

**Case report**

Patient information and medical history

A female which is 59 years old. In 2016, CT showed dense nodule shadow, high-density lock strip shadow and fuzzy patch shadow in the left lower lung, and focal thickening of the left pleura. Therefore, "left chest wall tumor resection + left lower lung tumor resection" was performed. Pathological report showed that low to moderate malignant fibrosarcoma (fibromyxoid type). In 2018, a peanut sized tumor was found on the left chest wall, which was hard without tenderness and untreated. CT Reexamination in 2020 showed multiple solid nodules in both lungs with clear boundary; The left chest wall is a kind of round mass shadow, which seems to have a pedicle connected with the rear muscle and is unevenly strengthened. It increased like an egg within one year. The CT re-examination in 2021 showed that there were multiple solid nodules in both lungs and tumors on the left chest wall, and the tumors on the left chest wall were significantly larger than before. No abnormality was found in other examinations.

Gross morphology and pathological diagnosis

The tumor is complete, the boundary is clear, the size is 6x5x4cm, the section is gray and white, and the local part is yellow and white . (**Figure 1**). Although the tumor generally seems to have a clear boundary, it infiltrates into adjacent tissues under microscope. At low magnification, we can see that the tumor is composed of alternating collagen like and mucinous areas. There is migration or transition between the two areas, and we can also see a relatively clear boundary. At high magnification, the tumor cells are consistent in morphology, spindle or short spindle, and star shaped in the myxoid area, similar to fibroblasts. The nucleus is round or oval, deeply stained, and the chromatin is evenly distributed. The mitotic nucleus is not obvious; The cytoplasm was lightly stained and the cell boundary was unclear. The tumor cells are arranged in bundles, linear arrangement or disorderly distribution. The blood vessels in tumor cells are relatively rare, mostly arched, curved or arc-shaped. In myxoid areas, sometimes branching capillary networks similar to myxoid liposarcoma can be seen. In the local area, the density of tumor cells increased significantly, mixed with collagen fibers, and small focal sclerosing epithelioid fibrosarcoma appeared (**Figure 2**).

Immunohistochemical results showed that SMA, Desmin, CD34, STAT6, S100, SOX10, HMB45 and Melan A were negative, EMA was weakly positive, MUC4 was diffuse and strongly positive, and Ki-67 index was low (3%). FISH results showed that DDIT3 gene mutation was not found, but FUS gene was break mutation(**Figure 3**).

A B


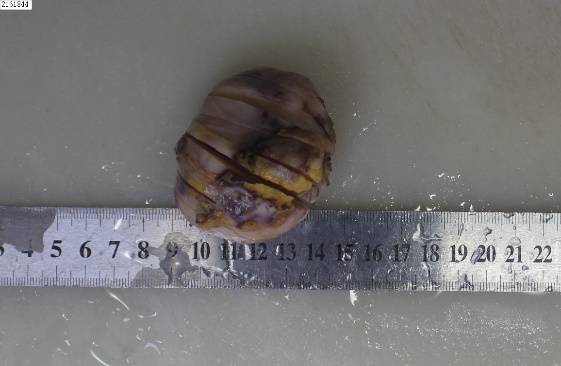

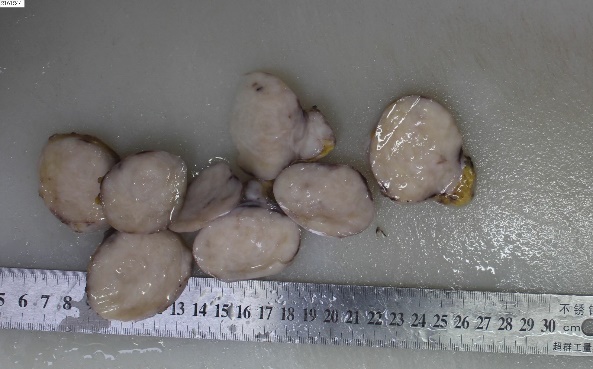


**Figure 1** The boundary of chest wall tumor is clear(A)and the section is grayish white and locally grayish yellow(B).

A B


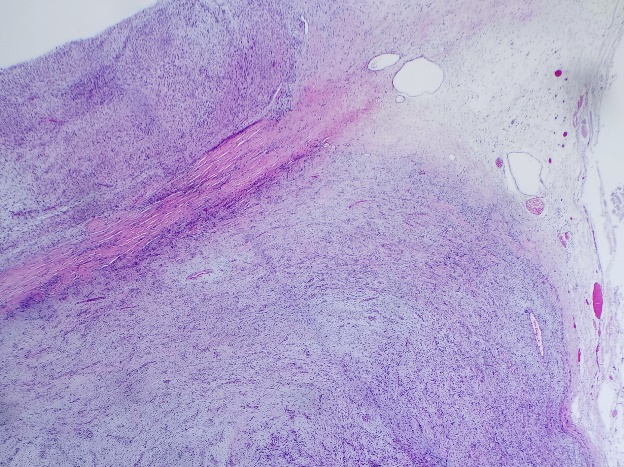

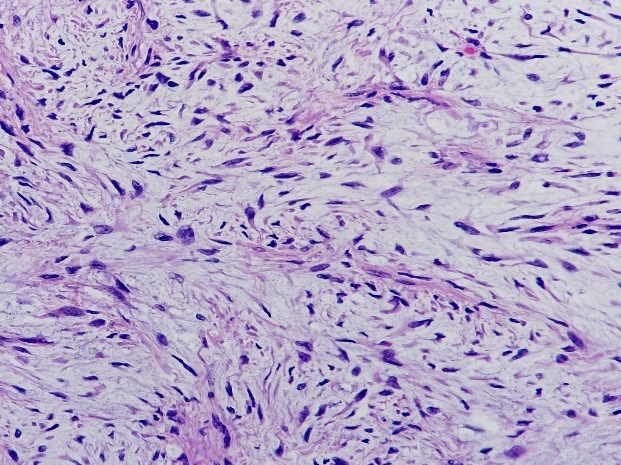


C D


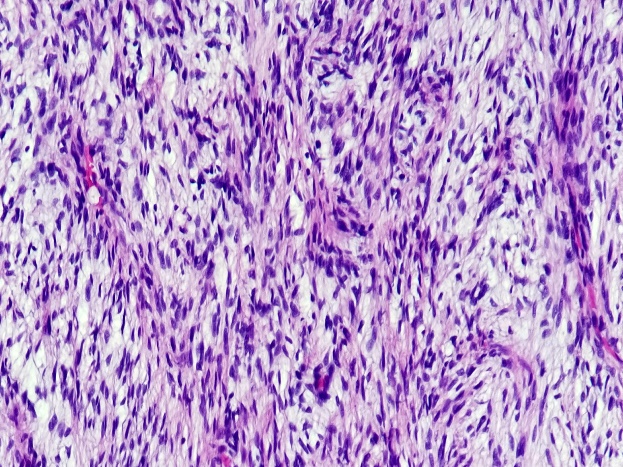

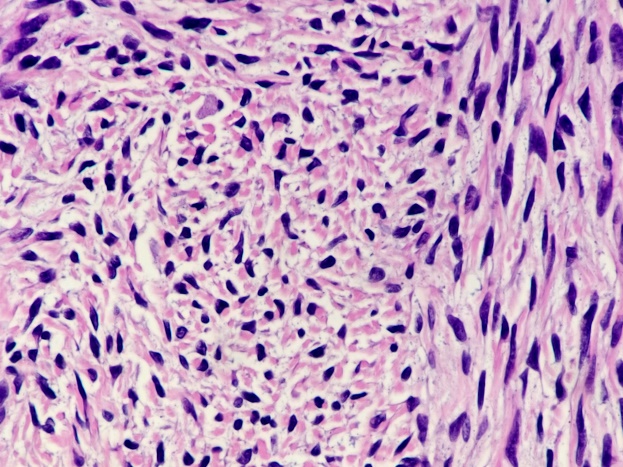


**Figure 2** LGFM appears to be encapsulated and slightly leafy at low magnification(A). At high magnification, mixed migration of collagen like area and mucoid area(B), tumor cells are consistent in morphology, showing spindle or short spindle shape(C). SEF appeared in small foci(D).

A B

**
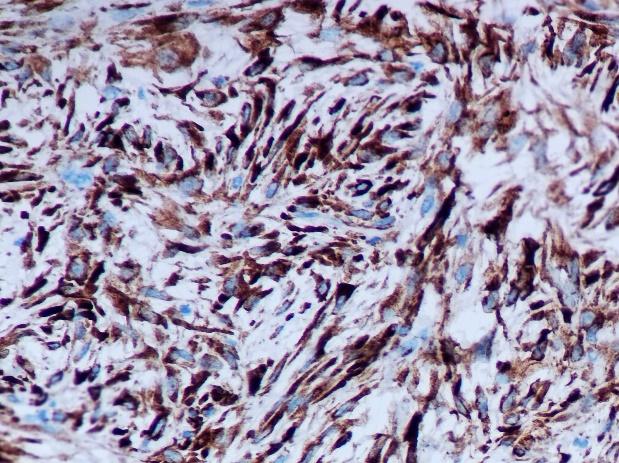

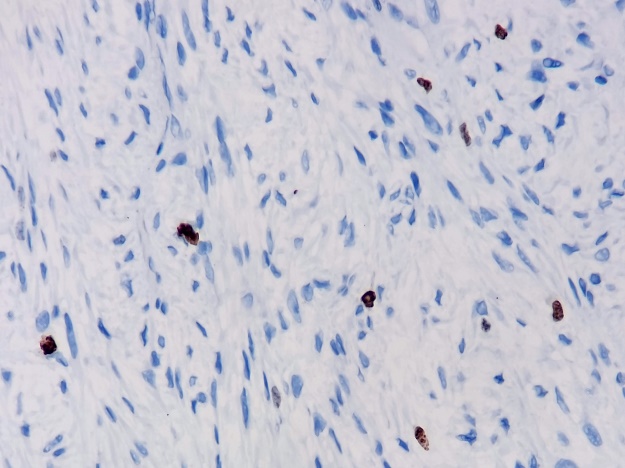
**

C D


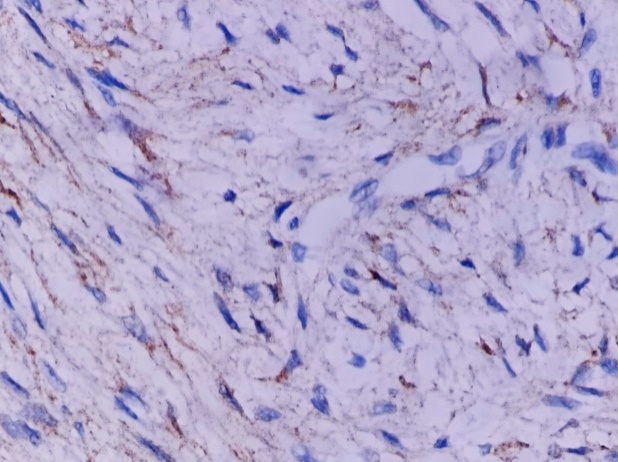

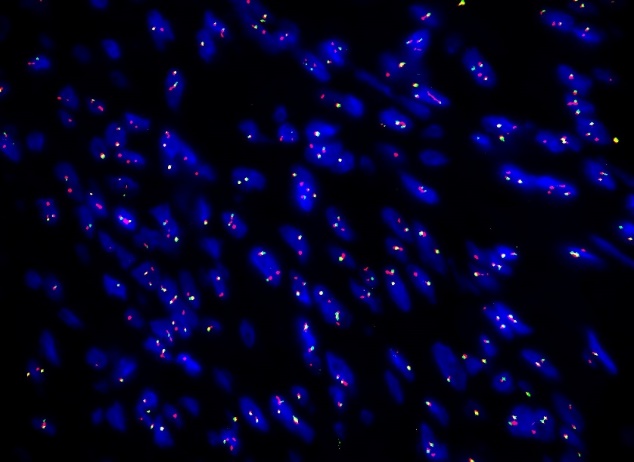


**Figure 3** Immunohistochemistry show MUC4 diffuse strong positive(A), Ki67 index low(B) and EMA Focal positive(C). FISH results FUS gene break mutation(D).

Next-generation sequencing technology

In order to find out whether there are mutations in other genes in low-grade fibromyxoid sarcoma except FUS-CREB3L2 fusion, FUS-CREB3L1 and EWSR1- CREB3L1 fusion [4, 15, 18, 19]. Subsequently, we conducted next-generation sequencing experiment, which showed that there were mutation in four gene MET, EGFR, KMT2B and RET (**Table 1**). It is noteworthy that KMT2B mutation in Low-grade fibromyxoid sarcoma has not been reported.

KMT2B belongs to a member of the histone lysine N-methyltransferase 2 family[26]. KMT2, also known as mixed lineage leukemia (MLL), is divided into KMT2A, KMT2B, KMT2C, KMT2D, KMT2F, KMT2G, etc. of which the two have high homology (KMT2A and KMT2B, KMT2C and KMT2D, KMT2F and KMT2G). The first four are the most common gene changes in tumor types, among which the mutation rate is higher in melanoma, Endometrial carcinoma and lung cancer. The mutation frequencies of single genes in KMT2 family are KMT2D(18%), KMT2C(15%), KMT2A(9%)and KMT2B(8%)[27].

KMT2B, also known as mixed lineage leukemia 2(MLL2), OMIM 606834, is located on chromosome 19q13 12. It is composed of 2715 amino acids and expressed in most tissues[28], which has a similar gene structure to MLL1(KMT2A)located on chromosome 11q23[27]. The structural components of the gene include the catalytically active C-terminal SET domain, a CXXC domain, an AT hook and several plant homeotic domains(PHD)in the N-terminal region[27, 29]. The SET domain binds to methyltransferase cofactor S-adenosylmethionine and the N-terminal tail of histone H3 catalyzing the methylation reaction[30], The CXXC domain composed of two zinc ions and four cysteine residues(Cys4),also known as the Zinc-finger(ZF)-CXXC domain, recognizes and binds to non-methylated CpG DNA, being critical for the association of MLL2 to chromatin[31],CXXC recognizes CpG islands[32]of most promoters, and PDH is next to zinc finger(ZF)-CXXC, including PDH1-PDH4[33], PDH possess a Cys4-His-Cys3 motif, coordinated by two zinc ions and mediating binding to methylated histone H3[34], Although all MLL families contain PDH, PDH3 of MLL2 show different specificity, which mainly binds to H3K4me3 tails[34].The C-terminal SEF domain of KMT2B forms a complex with WRAD(WDR5, RbBP5, ASH2L, ASH2L), host cell factors 1/2(HCF 1/2)and Menin[30, 35-37], the complex is responsible for the binding of PDH3 and H3K4me3, regulating bivalent developmental genes as well as stem cell and germinal cell Differentiation gene sets[32].In addition, MLL2 plays a key role in embryonic development, the deletion of MLL2 is related to early growth retardation, neural tube defects and apoptosis leading to embryonic death, it is also involved in the control of autonomic movement and the pathogenesis of dystonia in early children[38-40]. At the same time, MLL2 also has cancer promoting effects, including in colorectal cancer[41], gastric cancer[42], glioblastoma [43], etc.

Epidermal growth factor receptor (EGFR, also known as ErbB1 or HER-1) is a transmembrane glycoprotein consisting of an extracellular ligand-binding domain and a cytoplasmic tyrosine kinase domain. EGFR belongs to a family of four related receptor tyrosine kinases that play key mediators in cell signaling pathways such as proliferation, apoptosis, angiogenesis and metastasis[44] . It has been reported that FUS contains two targeted EGFR phosphorylation sites, mainly Y6 and Y304 in the FUS, suggesting that FUS can be phosphorylated by EGFR, which promoting FUS phosphorylation and inducing nuclear translocation through activated EGFR, so that FUS can mediate collagen production and creates a collagenous background. Therefore, EGFR-mediated FUS phosphorylation regulates FUS nuclear translocation and promotes transcription of fibrotic collagen genes [22].

| **Tumor specific mutation** |
| --- |

Gene variation mutant

| MET Copy number amplification - |
| --- |
| EGFR Copy number amplification - |
| KMT2B Missense mutation in exon 28 of p.F2163Y c.6488T>A (p.F2163Y)  RET Missense mutation in exon 20 of p.D1113G c.3338A>G (p.D1113G) |

**Table 1** The result of Next-generation sequencing.

**Conclusion**

Our case is the first to report the mutation of KMT2B gene in MUC4 diffusely positive low-grade fibromyxoid sarcoma. The tumor morphology is easy to be confused with low-grade malignant myxofibrosarcoma, invasive fibromatosis and sclerosing epithelioid fibrosarcoma, but the current view is that LGFM and SEF may belong to the same source. At low magnification, the tumor is composed of alternating collagen like and mucinous regions, there is migration or transition between the two regions. At the same time, MCU4 is diffuse and strongly positive. It is reported that most LGFM have FUS-CREB3L2 fusion [15-17].However, we sequenced the case and found mutations in KMT2B, MET, EGFR and RET gene, while no mutations in KMT2B were reported in LGFM before. Meanwhile, it has been reported that EGFR can promote FUS phosphorylation and nuclear translocation, leading to the production of fibrotic collagen, while under microscope, LGFM shows the distribution of gelatinous and myxoid regions alternately which may be related to the simultaneous mutation of these two genes, it needs to be proved later. Meanwhile, the mutation of KMT2B may be a new mutation point of LGFM, which may provide help for the diagnosis and treatment of LGFM.

**Discussison**

LGFM is a rare fibrosarcoma, which is mainly found in the limbs and trunk [4], but it also occurs occasionally in the scrotum, mediastinum, head and neck[5, 6, 8], while we report that it occurs occasionally in the chest wall and lung. The tumor is mainly composed of alternating collagen like and mucinous areas. The cells are spindle shaped, the cell density is not high, and it seems mild. In fact, the recurrence and distant metastasis rate is high. It needs to be distinguished from spindle cell tumors and tumors with fibromyxoid stroma, such as low-grade myxofibrosarcoma, neurofibroma, protuberant cutaneous fibrosarcoma, etc.[1, 45].The difference between the disease and others. Firstly, The alternating distribution of fibrous and myxoid areas. Secondly, MUC4 was diffuse and strongly positive. Thirdly, Chromosome translocation, the most commonFUS-CREB3L2 fusion. This case is easy to be mixed with low-grade myxofibrosarcoma because of their similar morphology. LGFM has basically no expression except MUC4 diffuse strong positive, but low-grade myxofibrosarcoma will have DDIT3 gene breakage, while LGFM has MUC4 strong positive expression and FUS-CREB3L2 fusion.

MUC4 is currently considered to be the most specific and sensitive indicator of LGFM. It is diffusely and strongly positive in LGFM, but it is also reported in the literature that MUC4 of LGFM is negative,FUS-CREB3L2 fusion exists at this time [25]. The tumor is also difficult to distinguish from SEF. They are considered to be homologous[46], and the positive expression rate of MUC4 is very high. However, there are also MUC4 negative cases in SEF. YAP1-KMT2A fusion and KMT2A-VIM fusion exist in SEF with MUC4 negative [47].We mentioned earlier that KMT2A and KMT2B belong to the same family, and they have high homology. However, we found the mutation of KMT2B gene in the sequencing of this case, and there are a few other gene fusion in LGFM, which needs to be further verified.

It has been previously reported that EGFR activation can promote FUS phosphorylation and nuclear translocation , and promote transcription of fibrotic collagen genes [22], and the LGFM showed gelatinous and myxoid regions under microscope, at the same time, our result of sequencing and FISH found mutations in both EGFR and FUS, so we speculated that the collagenous background may be related to mutations in EGFR and FUS. Since FUS-CREB3L2 fusion occurs after FUS fracture[18] , it cannot be ruled out that FUS mutant fracture may refuse with KMT2B or EGFR or other genes, and this needs to be further proved.

In conclusion, we report a case of MUC4 positive low-grade fibromyxoid sarcoma with KMT2B mutation in the chest wall. The tumor also has focal SEF, but the tumor has unique histological morphology, specific immunohistochemistry and chromosome translocation. LGFM seems mild, and local recurrence and distant metastasis are still very high. However, Our sequencing found that there is a mutation of KMT2B gene in LGFM. It is necessary to find out the fusion gene to provide further diagnosis and treatment for the tumor.

**Availability of data and materials**

The dataset supporting the conclusions of this article is included within the article.

**Abbreviations**

LGFM: Low-grade Fibromyxoid Sarcoma

SEF: Sclerosing Epithelioid Fibrosarcoma

CT: computed tomography

KMT2B: mixed lineage leukemia

EGFR: Epidermal growth factor receptor

MUC4: Mucin-4

MET: Mesenchymal-epithelial transition factor

RET: rearranged during transfection

FUS: Fused in Sarcoma

EMA: epithelial membrane antigen

**References**

1. Mohamed, M., C. Fisher, and K. Thway, *Low-grade fibromyxoid sarcoma: Clinical, morphologic and genetic features.* Ann Diagn Pathol, 2017. **28**: p. 60-67.

2. Maretty-Nielsen, K., et al., *Low-Grade Fibromyxoid Sarcoma: Incidence, Treatment Strategy of Metastases, and Clinical Significance of the FUS Gene.* Sarcoma, 2013. **2013**: p. 256280.

3. *HARRY L.EVANS.,M.D. A Report of Two Metastasizing Neoplasms Having a Deceptively Benign Appearance.* February 23,1987.

4. Mertens, F., et al., *Clinicopathologic and molecular genetic characterization of low-grade fibromyxoid sarcoma, and cloning of a novel FUS/CREB3L1 fusion gene.* Lab Invest, 2005. **85**(3): p. 408-15.

5. Chitayat, S., et al., *Case Report: An extremely rare occurrence of recurrent inguinal low-grade fibromyxoid sarcoma involving the scrotum.* F1000Res, 2020. **9**: p. 789.

6. Huang, J., S. Cohen, and G. Jour, *Primary small intestine mesenteric low-grade fibromyxoid sarcoma with foci of atypical epithelioid whorls and diffuse DOG1 expression: a case report.* Diagn Pathol, 2020. **15**(1): p. 23.

7. Cowan, M.L., et al., *Low-Grade Fibromyxoid Sarcoma of the Head and Neck: A Clinicopathologic Series and Review of the Literature.* Head Neck Pathol, 2016. **10**(2): p. 161-6.

8. Sajid, M.I., et al., *Low-grade fibromyxoid sarcoma incidentally discovered as an asymptomatic mediastinal mass: a case report and review of the literature.* J Med Case Rep, 2021. **15**(1): p. 50.

9. Harry L. Evans,M.D. *Low-Grade Fibromyxoid Sarcoma:A Report of 12 Cases.*Surgical Pathology,1993.17(6):595-600*.*

10. Andrew L., et al., *Low-Grade Fibromyxoid Sarcoma and Hyalinizing Spindle Cell Tumor With Giant Rosettes.* Surgical Pathol,2000.24(10):1353-1360*.*

11. GOODLAD,J.R., et al., *Low grade fibomyxoid szrcoma:clinicopathological analysis of eleven new cases in support of a distinct entity.Histopathology.* 1995.26,229-237*.*

12. Zamecnik, M. and M. Michal, *Low-grade fibromyxoid sarcoma: a report of eight cases with histologic, immunohistochemical, and ultrastructural study.* Ann Diagn Pathol, 2000. **4**(4): p. 207-17.

13. Steven D., et al., *Superficial Low-grade Fibromyxoid Sarcoma:A Clinicopathologic Analysis of 19 Cases With a Unique Observation in the Pediatric Population.* Am J Surg Pathol, 2005.29:24-210.*.*

14. Le Cesne, A., et al., *A retrospective analysis of antitumour activity with trabectedin in translocation-related sarcomas.* Eur J Cancer, 2012. **48**(16): p. 3036-44.

15. Panagopoulos, I., et al., *The chimeric FUS/CREB3l2 gene is specific for low-grade fibromyxoid sarcoma.* Genes Chromosomes Cancer, 2004. **40**(3): p. 218-28.

16. Atsuji Matsuyama, MD., et al., *Molecular Detection of FUS-CREB3L2 Fusion Transcripts in Low-grade Fibromyxoid Sarcoma Using Formalin-fixed,Paraffin-embedded Tissue Specimens.* Am J surg Pathol, 2006. 30:1077-1084.*.*

17. Panagopoulos, I., et al., *Characterization of the native CREB3L2 transcription factor and the FUS/CREB3L2 chimera.* Genes Chromosomes Cancer, 2007. **46**(2): p. 181-91.

18. Storlazzi, C.T., et al., *Fusion of the FUS and BBF2H7 genes in low grade fibromyxoid sarcoma.* Hum Mol Genet, 2003. **12**(18): p. 2349-58.

19. Patrick P.L., et al., *EWSR1-CREB3L1 Gene Fusion:A Nover Alternative Molecular Aberration of Low-grade Fibromyxoid Sarcoma.* Am J Surg Pathol. 2013. 37:734-738*.*

20. Ederle, H. and D. Dormann, *TDP-43 and FUS en route from the nucleus to the cytoplasm.* FEBS Lett, 2017. **591**(11): p. 1489-1507.

21. Law, W.J., K.L. Cann, and G.G. Hicks, *TLS, EWS and TAF15: a model for transcriptional integration of gene expression.* Brief Funct Genomic Proteomic, 2006. **5**(1): p. 8-14.

22. Chiusa, M., et al., *EGF receptor-mediated FUS phosphorylation promotes its nuclear translocation and fibrotic signaling.* J Cell Biol, 2020. **219**(9).

23. Leoma A., er al.,*MUC4 is a Highly Sensitive and Specific Marker for Low-grade Fibromyxoid Sarcoma*.Am J Surg Pathol. 2011. 35:733-741*.*

24. Leoma A., er al., *MUC4 is a Sensitive and Exremely Useful Marker for Sclerosing Epithelioid Fibrosarcoma.*Am J Surg Pathol. 2012. 36:1444-1451*.*

25. Linos, K., J.A. Bridge, and M.A. Edgar, *MUC 4-negative FUS-CREB3L2 rearranged low-grade fibromyxoid sarcoma.* Histopathology, 2014. **65**(5): p. 722-4.

26. Zhang, P. and Y. Huang, *Genomic alterations in KMT2 family predict outcome of immune checkpoint therapy in multiple cancers.* J Hematol Oncol, 2021. **14**(1): p. 39.

27. Rao, R.C. and Y. Dou, *Hijacked in cancer: the KMT2 (MLL) family of methyltransferases.* Nat Rev Cancer, 2015. **15**(6): p. 334-46.

28. Kevin T., et al., *MLL2: A New Mammalian Member of the trx/MLL Family of Genes.*Genomics. 1999. 59,187-192*.*

29. Zhang, J., et al., *Germline Mutations in Predisposition Genes in Pediatric Cancer.* N Engl J Med, 2015. **373**(24): p. 2336-2346.

30. Li, Y., et al., *Structural basis for activity regulation of MLL family methyltransferases.* Nature, 2016. **530**(7591): p. 447-52.

31. Allen, M.D., et al., *Solution structure of the nonmethyl-CpG-binding CXXC domain of the leukaemia-associated MLL histone methyltransferase.* EMBO J, 2006. **25**(19): p. 4503-12.

32. Klonou, A., S. Chlamydas, and C. Piperi, *Structure, Activity and Function of the MLL2 (KMT2B) Protein Lysine Methyltransferase.* Life (Basel), 2021. **11**(8).

33. Ali, M., et al., *Diverse functions of PHD fingers of the MLL/KMT2 subfamily.* Biochim Biophys Acta, 2014. **1843**(2): p. 366-71.

34. Sanchez, R. and M.M. Zhou, *The PHD finger: a versatile epigenome reader.* Trends Biochem Sci, 2011. **36**(7): p. 364-72.

35. Patel, A., et al., *On the mechanism of multiple lysine methylation by the human mixed lineage leukemia protein-1 (MLL1) core complex.* J Biol Chem, 2009. **284**(36): p. 24242-56.

36. Cao, F., et al., *An Ash2L/RbBP5 heterodimer stimulates the MLL1 methyltransferase activity through coordinated substrate interactions with the MLL1 SET domain.* PLoS One, 2010. **5**(11): p. e14102.

37. Xue, H., et al., *Structural basis of nucleosome recognition and modification by MLL methyltransferases.* Nature, 2019. **573**(7774): p. 445-449.

38. Meyer, E., et al., *Mutations in the histone methyltransferase gene KMT2B cause complex early-onset dystonia.* Nat Genet, 2017. **49**(2): p. 223-237.

39. Zech, M., et al., *Haploinsufficiency of KMT2B, Encoding the Lysine-Specific Histone Methyltransferase 2B, Results in Early-Onset Generalized Dystonia.* Am J Hum Genet, 2016. **99**(6): p. 1377-1387.

40. Park, K., J.A. Kim, and J. Kim, *Transcriptional regulation by the KMT2 histone H3K4 methyltransferases.* Biochim Biophys Acta Gene Regul Mech, 2020. **1863**(7): p. 194545.

41. Sierra, J., et al., *The APC tumor suppressor counteracts beta-catenin activation and H3K4 methylation at Wnt target genes.* Genes Dev, 2006. **20**(5): p. 586-600.

42. Sai Ge., et al., *Genomic alterations in advanced gastric cancer endoscopic biopsy samples using targeted next-generation sequencing.* Am J Cancer Res, 2017.7(7):1540-1553*.*

43. Wong, W.H., et al., *NF1 glioblastoma clonal profiling reveals KMT2B mutations as potential somatic oncogenic events.* Neurology, 2019. **93**(24): p. 1067-1069.

44. Ayati, A., et al., *A review on progression of epidermal growth factor receptor (EGFR) inhibitors as an efficient approach in cancer targeted therapy.* Bioorg Chem, 2020. **99**: p. 103811.

45. Alfaro-Cervelló, C., et al., *Sarcoma fibromixoide de bajo grado, un diagnóstico diferencial esencial en los tumores mixoides de apariencia benigna.* Revista Española de Patología, 2018. **51**(3): p. 178-182.

46. Prieto-Granada, C., et al., *A genetic dichotomy between pure sclerosing epithelioid fibrosarcoma (SEF) and hybrid SEF/low-grade fibromyxoid sarcoma: a pathologic and molecular study of 18 cases.* Genes Chromosomes Cancer, 2015. **54**(1): p. 28-38.

47. Kao, Y.C., et al., *Recurrent YAP1 and KMT2A Gene Rearrangements in a Subset of MUC4-negative Sclerosing Epithelioid Fibrosarcoma.* Am J Surg Pathol, 2020. **44**(3): p. 368-377.

**Acknowledgements**

Not applicable.

**Funding**

No funding.

**Author information**

Affiliations

**Department of Pathology, Guangdong Provincial People’s Hospital, Guangdong Academy of Medical Sciences, Guangzhou, China**

Contributions

Liying Zhang; Luqiao Luo; Chao Liu; Zhi Li: Manuscript editing. The author(s) read and approved the final manuscript.

Corresponding author

Correspondence to Zhi Li.

**Ethics declarations**

Ethics approval and consent to participate

All ethical approval and consent procedures were approved by Ethies Review Committee of Guangdong Provincial People’s Hospital.

Consent for publication

Not applicable.

Competing interests

The authors declare no conflict of interest/competing interests in publishing the present manuscript.
